# *catena*-Poly[[[[3-(2-pyrid­yl)-1*H*-pyra­zole]nickel(II)]-μ-oxalato] sesquihydrate]

**DOI:** 10.1107/S1600536809026117

**Published:** 2009-07-11

**Authors:** Bin Jiang, Zhilu Liu

**Affiliations:** a Department of Pharmacy, Shandong Medical College, Jinan 250002, People’s Republic of China; b State Key Laboratory of Solid Lubrication, Lanzhou Institute of Chemical Physics, Chinese Academy of Sciences, Lanzhou 73000, People’s Republic of China

## Abstract

In the title compound, {[Ni(C_2_O_4_)(C_8_H_7_N_3_)]·1.5H_2_O}_*n*_, both unique Ni^II^ ions are chelated by an *O*,*O*′-bidentate oxalate ion and an *N*,*N*′-bidentate 3-(2-pyrid­yl)pyrazole mol­ecule. A second, symmetry-generated, oxalate ion completes a distorted *cis*-NiN_2_O_4_ octa­hedral geometry for both metal centres. The bridging oxalate ions result in two distinct wave-like polymeric chains propagating in [100]. The packing is consolidated by N—H⋯O and O—H⋯O hydrogen bonds. The crystal studied was found to be an inversion twin.

## Related literature

For related literature on coordination polymers, see: Ward (2007 ▶).
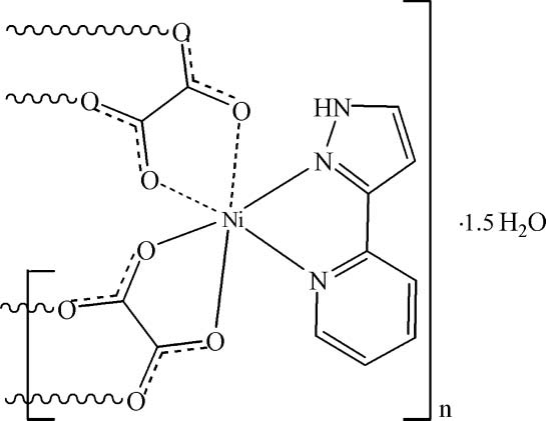


## Experimental

### 

#### Crystal data

[Ni(C_2_O_4_)(C_8_H_7_N_3_)]·1.5H_2_O
*M* *_r_* = 318.92
Orthorhombic, 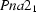

*a* = 9.763 (2) Å
*b* = 9.1970 (18) Å
*c* = 29.352 (6) Å
*V* = 2635.5 (9) Å^3^
*Z* = 8
Mo *K*α radiation
μ = 1.50 mm^−1^
*T* = 293 K
0.12 × 0.10 × 0.08 mm

#### Data collection

Bruker APEXII CCD area-detector diffractometer
Absorption correction: multi-scan (*SADABS*; Bruker, 2004 ▶) *T*_min_ = 0.841, *T*_max_ = 0.890
17815 measured reflections
4882 independent reflections
3723 reflections with *I* > 2σ(*I*)
*R*_int_ = 0.059

#### Refinement

*R*[*F* ^2^ > 2σ(*F* ^2^)] = 0.056
*wR*(*F* ^2^) = 0.139
*S* = 1.00
4882 reflections
385 parameters
12 restraints
H atoms treated by a mixture of independent and constrained refinement
Δρ_max_ = 0.47 e Å^−3^
Δρ_min_ = −0.37 e Å^−3^
Absolute structure: Flack (1983 ▶), 2287 Friedel pairs
Flack parameter: 0.50 (3)

### 

Data collection: *APEX2* (Bruker, 2004 ▶); cell refinement: *SAINT-Plus* (Bruker, 2004 ▶); data reduction: *SAINT-Plus*; program(s) used to solve structure: *SHELXS97* (Sheldrick, 2008 ▶); program(s) used to refine structure: *SHELXL97* (Sheldrick, 2008 ▶); molecular graphics: *SHELXTL* (Sheldrick, 2008 ▶); software used to prepare material for publication: *SHELXTL*.

## Supplementary Material

Crystal structure: contains datablocks global, I. DOI: 10.1107/S1600536809026117/hb5014sup1.cif

Structure factors: contains datablocks I. DOI: 10.1107/S1600536809026117/hb5014Isup2.hkl

Additional supplementary materials:  crystallographic information; 3D view; checkCIF report

## Figures and Tables

**Table 1:** Selected bond lengths (Å)

Ni1—O1	2.152 (5)
Ni1—O3	2.200 (6)
Ni1—O2^i^	2.163 (6)
Ni1—O4^i^	2.177 (4)
Ni1—N1	2.259 (6)
Ni1—N2	2.230 (6)
Ni2—O6	2.159 (6)
Ni2—O8	2.188 (4)
Ni2—O5^ii^	2.142 (4)
Ni2—O7^ii^	2.217 (6)
Ni2—N4	2.285 (6)
Ni2—N5	2.205 (6)

Symmetry codes: (i) 
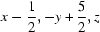
; (ii) 
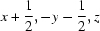
.

**Table 2:** Hydrogen-bond geometry (Å, °)

*D*—H⋯*A*	*D*—H	H⋯*A*	*D*⋯*A*	*D*—H⋯*A*
N3—H3*A*⋯O11^iii^	0.97 (4)	1.81 (2)	2.745 (10)	160 (5)
N6—H6*A*⋯O10^iv^	0.98 (16)	2.0 (2)	2.732 (9)	133 (5)
O9—H2*W*⋯O7^v^	0.82 (4)	2.02 (4)	2.822 (9)	166 (6)
O9—H1*W*⋯O11^vi^	0.82 (12)	2.22 (15)	2.813 (11)	130 (16)
O10—H3*W*⋯O4^vii^	0.82 (5)	2.30 (7)	2.834 (9)	123 (7)
O10—H4*W*⋯O9^vi^	0.82 (4)	2.08 (3)	2.811 (11)	148 (5)
O11—H5*W*⋯O9^viii^	0.82 (5)	2.15 (4)	2.813 (11)	138 (5)
O11—H6*W*⋯O8^ix^	0.82 (4)	2.17 (4)	2.876 (9)	144 (6)

Symmetry codes: (iii) 
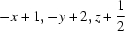
; (iv) 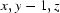; (v) 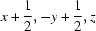; (vi) 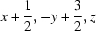; (vii) 
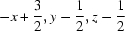
; (viii) 
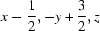
; (ix) 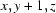.

**Fig. 1.**
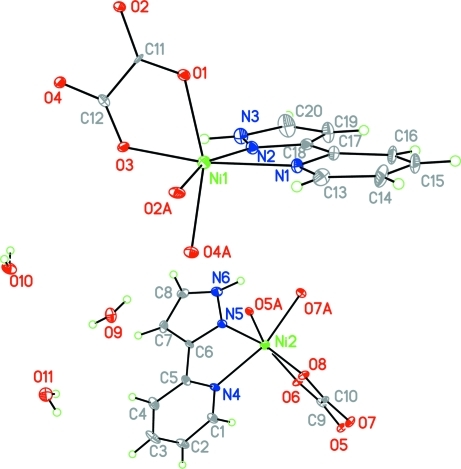
A view of (I) with the unique atoms labelled. Displacement ellipsoids are drawn at the 30% probability level.

**Fig. 2.**
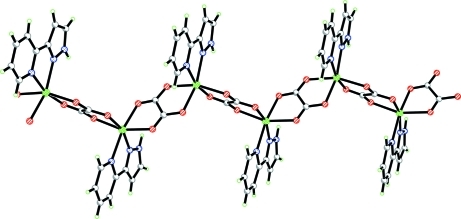
A view of the chain strcuture of (I).

**Fig. 3.**
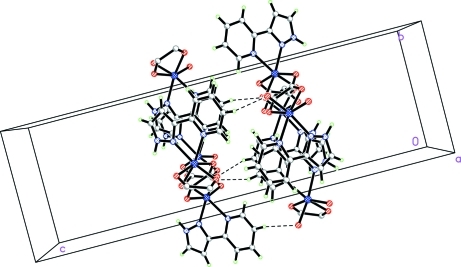
A view of the packing strcuture of (I).

## supplementary crystallographic information

### Comment

As part of our ongoing studies of coordination polymers (Ward, 2007), we now report the synthesis and crystal structural characterization of the title compound, (I).

The N^II^ ions are hexcoordianted, chelated by two oxalate and one 3-(2-pyridyl)pyrazole ligand (Table 1). While each oxalate ligand acts as one bridige to chalate two Ni ions, forming one wave-like line with Ni···Ni distance being 5.562 Å, shown in Figure 2. The structure is consolidated by N—H···O and O—H···O hydrogen bonds (Table 2, Figure 3).

### Experimental

The synthesis was performed in a 25 ml Teflon-lined stainless steel vessel: NiCl_2_ (1 mmol), 3-(2-pyridyl)pyrazole (1 mmol), oxalic acid (1 mmol), and H_2_O (10 ml) were mixed and heated to 433 K for three days. On cooling, green blocks of (I) were recovered. Anal. Calc. for C_20_H_20_Ni_2_N_6_O_11_: C 37.60, H 3.13, N 13.16%; Found: 37.56, H 3.06, N 13.10%.

### Refinement

The C-bound H atoms were geometrically planced (C—H = 0.93Å) and refined as riding with *U*_iso_ = 1.2*U*_eq_(C).  The N- and O-bound H atoms were located in difference maps and refined with distance restraints: N—H = 0.97 (1)Å, O—H = 0.82 (2)Å, H···H = 1.38 (2)Å.

### Crystal data

**Table:**

[Ni(C_2_O_4_)(C_8_H_7_N_3_)]·1.5H_2_O	*F*(000) = 1304
*M**_r_* = 318.92	*D*_x_ = 1.607 Mg m^−^^3^
Orthorhombic, *P**n**a*2_1_	Mo *K*α radiation, λ = 0.71073 Å
Hall symbol: P 2c -2n	Cell parameters from 4779 reflections
*a* = 9.763 (2) Å	θ = 2.3–25.5°
*b* = 9.1970 (18) Å	µ = 1.50 mm^−^^1^
*c* = 29.352 (6) Å	*T* = 293 K
*V* = 2635.5 (9) Å^3^	Block, green
*Z* = 8	0.12 × 0.10 × 0.08 mm

### Data collection

**Table:**

Bruker APEXII CCD area-detector diffractometer	4882 independent reflections
Radiation source: fine-focus sealed tube	3723 reflections with *I* > 2σ(*I*)
graphite	*R*_int_ = 0.059
φ and ω scans	θ_max_ = 25.5°, θ_min_ = 2.3°
Absorption correction: multi-scan (*SADABS*; Bruker, 2004)	*h* = −11→11
*T*_min_ = 0.841, *T*_max_ = 0.890	*k* = −11→10
17815 measured reflections	*l* = −35→35

### Refinement

**Table:**

Refinement on *F*^2^	Secondary atom site location: difference Fourier map
Least-squares matrix: full	Hydrogen site location: inferred from neighbouring sites
*R*[*F*^2^ > 2σ(*F*^2^)] = 0.056	H atoms treated by a mixture of independent and constrained refinement
*wR*(*F*^2^) = 0.139	*w* = 1/[σ^2^(*F*_o_^2^) + (0.078*P*)^2^ + 0.5477*P*] where *P* = (*F*_o_^2^ + 2*F*_c_^2^)/3
*S* = 1.00	(Δ/σ)_max_ = 0.001
4882 reflections	Δρ_max_ = 0.47 e Å^−^^3^
385 parameters	Δρ_min_ = −0.36 e Å^−^^3^
12 restraints	Absolute structure: Flack (1983), 2287 Friedel pairs
Primary atom site location: structure-invariant direct methods	Flack parameter: 0.50 (3)

### Special details

**Table:**

Geometry. All e.s.d.'s (except the e.s.d. in the dihedral angle between two l.s. planes) are estimated using the full covariance matrix. The cell e.s.d.'s are taken into account individually in the estimation of e.s.d.'s in distances, angles and torsion angles; correlations between e.s.d.'s in cell parameters are only used when they are defined by crystal symmetry. An approximate (isotropic) treatment of cell e.s.d.'s is used for estimating e.s.d.'s involving l.s. planes.
Refinement. Refinement of *F*^2^ against ALL reflections. The weighted *R*-factor *wR* and goodness of fit *S* are based on *F*^2^, conventional *R*-factors *R* are based on *F*, with *F* set to zero for negative *F*^2^. The threshold expression of *F*^2^ > σ(*F*^2^) is used only for calculating *R*-factors(gt) *etc*. and is not relevant to the choice of reflections for refinement. *R*-factors based on *F*^2^ are statistically about twice as large as those based on *F*, and *R*- factors based on ALL data will be even larger.

### Fractional atomic coordinates and isotropic or equivalent isotropic displacement parameters (Å^2^)

**Table:**

	*x*	*y*	*z*	*U*_iso_*/*U*_eq_	
Ni1	0.31148 (9)	1.09616 (9)	0.84957 (7)	0.0406 (2)	
Ni2	0.55514 (8)	−0.09515 (9)	0.08796 (7)	0.0408 (2)	
C1	0.3921 (9)	0.0950 (8)	0.0119 (3)	0.049 (2)	
H1	0.3731	0.0049	−0.0010	0.059*	
C2	0.3379 (10)	0.2187 (11)	−0.0082 (4)	0.062 (2)	
H2	0.2809	0.2111	−0.0335	0.074*	
C3	0.3688 (12)	0.3506 (12)	0.0094 (3)	0.077 (4)	
H3	0.3325	0.4349	−0.0033	0.092*	
C4	0.4564 (11)	0.3578 (10)	0.0472 (4)	0.060 (3)	
H4	0.4850	0.4472	0.0585	0.073*	
C5	0.4996 (10)	0.2304 (8)	0.0671 (3)	0.041 (2)	
C6	0.5898 (9)	0.2314 (8)	0.1082 (3)	0.036 (2)	
C7	0.6452 (10)	0.3438 (9)	0.1323 (4)	0.062 (3)	
H7	0.6355	0.4430	0.1270	0.075*	
C8	0.7179 (10)	0.2767 (11)	0.1658 (4)	0.061 (3)	
H8	0.7664	0.3233	0.1888	0.073*	
C9	0.3240 (8)	−0.2826 (8)	0.0649 (3)	0.0342 (19)	
C10	0.2918 (7)	−0.2229 (7)	0.1133 (3)	0.0254 (15)	
C11	0.5767 (6)	1.2152 (8)	0.8712 (2)	0.0236 (13)	
C12	0.5476 (8)	1.2747 (8)	0.8228 (3)	0.0325 (18)	
C13	0.1336 (9)	0.9194 (11)	0.9256 (3)	0.057 (2)	
H13	0.1073	1.0111	0.9357	0.069*	
C14	0.0855 (11)	0.7996 (12)	0.9485 (3)	0.071 (3)	
H14	0.0292	0.8096	0.9738	0.085*	
C15	0.1225 (10)	0.6663 (12)	0.9331 (4)	0.070 (3)	
H15	0.0937	0.5844	0.9490	0.084*	
C16	0.1990 (10)	0.6493 (10)	0.8960 (4)	0.060 (3)	
H16	0.2158	0.5570	0.8843	0.072*	
C17	0.2534 (9)	0.7723 (8)	0.8749 (3)	0.0372 (19)	
C18	0.3377 (9)	0.7695 (8)	0.8356 (3)	0.040 (2)	
C19	0.3938 (12)	0.6511 (11)	0.8106 (4)	0.071 (3)	
H19	0.3789	0.5526	0.8157	0.085*	
C20	0.4713 (14)	0.7090 (12)	0.7784 (5)	0.088 (4)	
H20	0.5256	0.6582	0.7579	0.106*	
N1	0.2170 (6)	0.9107 (6)	0.8890 (2)	0.0378 (15)	
N2	0.3773 (6)	0.8926 (7)	0.8146 (2)	0.0384 (15)	
N3	0.4576 (7)	0.8577 (8)	0.7808 (2)	0.0516 (18)	
N4	0.4710 (6)	0.1009 (6)	0.0492 (2)	0.0353 (14)	
N5	0.6263 (6)	0.1013 (7)	0.1239 (2)	0.0375 (14)	
N6	0.7085 (8)	0.1366 (9)	0.1604 (2)	0.0536 (19)	
O1	0.4923 (5)	1.1355 (5)	0.88962 (16)	0.0351 (10)	
O2	0.6957 (5)	1.2463 (5)	0.8884 (3)	0.0398 (17)	
O3	0.4389 (5)	1.2387 (5)	0.8069 (2)	0.0347 (16)	
O4	0.6367 (5)	1.3553 (5)	0.80613 (16)	0.0354 (10)	
O5	0.2318 (4)	−0.3592 (5)	0.04713 (16)	0.0335 (10)	
O6	0.4326 (5)	−0.2429 (5)	0.0479 (3)	0.0378 (17)	
O7	0.1787 (6)	−0.2608 (6)	0.1324 (2)	0.0385 (17)	
O8	0.3784 (4)	−0.1410 (5)	0.13145 (17)	0.0383 (11)	
O9	0.6278 (8)	0.6448 (6)	0.2198 (2)	0.0605 (12)	
O10	0.8552 (8)	0.9517 (8)	0.2145 (2)	0.0727 (17)	
O11	0.3970 (8)	0.9636 (7)	0.2236 (2)	0.0667 (16)	
H6W	0.412 (4)	0.905 (5)	0.2033 (15)	0.077 (17)*	
H2W	0.632 (6)	0.689 (6)	0.1956 (11)	0.074 (19)*	
H1W	0.672 (16)	0.569 (10)	0.221 (3)	0.083 (10)*	
H3W	0.860 (5)	0.989 (8)	0.2399 (12)	0.085 (2)*	
H4W	0.927 (3)	0.918 (7)	0.2051 (17)	0.093 (2)*	
H5W	0.321 (4)	0.957 (7)	0.235 (3)	0.107 (4)*	
H3A	0.490 (5)	0.935 (4)	0.7611 (14)	0.064 (13)*	
H6A	0.71 (3)	0.069 (18)	0.186 (5)	0.059 (13)*	

### Atomic displacement parameters (Å^2^)

**Table:**

	*U*^11^	*U*^22^	*U*^33^	*U*^12^	*U*^13^	*U*^23^
Ni1	0.0366 (4)	0.0418 (5)	0.0432 (5)	−0.0004 (4)	0.0012 (4)	0.0039 (5)
Ni2	0.0352 (4)	0.0420 (5)	0.0453 (5)	0.0012 (4)	−0.0004 (4)	−0.0020 (5)
C1	0.064 (5)	0.039 (4)	0.045 (4)	0.017 (4)	−0.007 (4)	−0.010 (3)
C2	0.070 (6)	0.072 (6)	0.044 (5)	0.024 (5)	−0.019 (4)	−0.002 (5)
C3	0.116 (9)	0.074 (7)	0.041 (5)	0.056 (6)	−0.014 (5)	0.004 (5)
C4	0.092 (7)	0.037 (5)	0.053 (5)	0.014 (4)	−0.001 (5)	−0.008 (4)
C5	0.047 (5)	0.039 (4)	0.039 (5)	0.016 (4)	0.012 (4)	0.007 (3)
C6	0.043 (4)	0.030 (4)	0.035 (5)	0.005 (3)	−0.016 (3)	0.005 (3)
C7	0.081 (6)	0.024 (4)	0.082 (7)	−0.015 (4)	−0.021 (6)	−0.010 (4)
C8	0.062 (5)	0.057 (5)	0.062 (6)	−0.012 (5)	−0.037 (5)	−0.015 (5)
C9	0.051 (5)	0.024 (3)	0.028 (4)	0.025 (4)	0.000 (3)	−0.011 (3)
C10	0.016 (3)	0.031 (3)	0.029 (4)	−0.002 (3)	0.002 (3)	0.000 (3)
C11	0.016 (3)	0.027 (3)	0.028 (3)	−0.016 (3)	−0.002 (2)	−0.002 (3)
C12	0.037 (5)	0.031 (3)	0.029 (4)	0.010 (3)	0.005 (3)	0.007 (3)
C13	0.060 (5)	0.077 (6)	0.035 (4)	−0.011 (4)	0.008 (4)	−0.001 (4)
C14	0.095 (7)	0.082 (7)	0.035 (5)	−0.046 (7)	0.021 (5)	0.007 (5)
C15	0.082 (7)	0.055 (6)	0.071 (7)	−0.026 (5)	0.006 (5)	0.033 (5)
C16	0.065 (5)	0.033 (5)	0.083 (7)	−0.018 (4)	0.010 (5)	0.017 (5)
C17	0.044 (5)	0.031 (4)	0.038 (5)	−0.005 (3)	0.005 (4)	0.001 (3)
C18	0.036 (4)	0.031 (4)	0.051 (6)	0.000 (3)	−0.011 (4)	−0.004 (3)
C19	0.096 (8)	0.038 (5)	0.079 (7)	−0.002 (5)	0.024 (6)	0.006 (5)
C20	0.124 (10)	0.046 (5)	0.095 (10)	0.030 (7)	0.036 (8)	−0.023 (7)
N1	0.043 (3)	0.035 (4)	0.036 (3)	−0.002 (2)	0.006 (3)	0.004 (3)
N2	0.037 (3)	0.034 (3)	0.044 (3)	0.006 (2)	0.005 (3)	0.003 (3)
N3	0.050 (4)	0.047 (4)	0.058 (5)	0.009 (3)	0.019 (3)	−0.001 (3)
N4	0.035 (3)	0.037 (3)	0.034 (3)	0.016 (2)	−0.006 (2)	−0.007 (3)
N5	0.041 (3)	0.036 (3)	0.036 (3)	0.000 (3)	−0.014 (3)	−0.004 (3)
N6	0.061 (4)	0.058 (5)	0.042 (4)	−0.005 (3)	−0.027 (3)	0.000 (3)
O1	0.034 (2)	0.043 (3)	0.029 (2)	−0.001 (2)	−0.004 (2)	0.009 (2)
O2	0.042 (4)	0.042 (4)	0.035 (4)	−0.009 (2)	−0.019 (3)	0.0142 (19)
O3	0.023 (3)	0.045 (4)	0.036 (4)	−0.0085 (19)	−0.005 (2)	0.012 (2)
O4	0.034 (2)	0.041 (3)	0.031 (2)	−0.009 (2)	−0.0058 (19)	0.008 (2)
O5	0.030 (2)	0.039 (2)	0.032 (2)	−0.006 (2)	0.006 (2)	−0.011 (2)
O6	0.022 (3)	0.056 (4)	0.036 (4)	−0.0070 (19)	0.006 (2)	−0.018 (2)
O7	0.035 (3)	0.054 (4)	0.026 (4)	−0.015 (2)	0.016 (2)	−0.008 (2)
O8	0.028 (2)	0.052 (3)	0.035 (3)	−0.005 (2)	0.003 (2)	−0.016 (2)
O9	0.090 (4)	0.057 (3)	0.034 (2)	−0.003 (4)	0.015 (2)	0.007 (3)
O10	0.078 (5)	0.096 (5)	0.043 (4)	0.011 (4)	−0.014 (4)	0.017 (4)
O11	0.084 (5)	0.071 (4)	0.045 (4)	−0.006 (4)	−0.011 (4)	−0.005 (3)

### Geometric parameters (Å, °)

**Table:**

Ni1—O1	2.152 (5)	C11—O2	1.299 (8)
Ni1—O3	2.200 (6)	C11—C12	1.550 (11)
Ni1—O2^i^	2.163 (6)	C12—O3	1.206 (10)
Ni1—O4^i^	2.177 (4)	C12—O4	1.243 (9)
Ni1—N1	2.259 (6)	C13—N1	1.349 (11)
Ni1—N2	2.230 (6)	C13—C14	1.374 (12)
Ni2—O6	2.159 (6)	C13—H13	0.9300
Ni2—O8	2.188 (4)	C14—C15	1.355 (16)
Ni2—O5^ii^	2.142 (4)	C14—H14	0.9300
Ni2—O7^ii^	2.217 (6)	C15—C16	1.330 (15)
Ni2—N4	2.285 (6)	C15—H15	0.9300
Ni2—N5	2.205 (6)	C16—C17	1.395 (11)
C1—N4	1.340 (11)	C16—H16	0.9300
C1—C2	1.386 (11)	C17—N1	1.385 (10)
C1—H1	0.9300	C17—C18	1.416 (13)
C2—C3	1.353 (14)	C18—N2	1.346 (10)
C2—H2	0.9300	C18—C19	1.424 (13)
C3—C4	1.401 (15)	C19—C20	1.321 (17)
C3—H3	0.9300	C19—H19	0.9300
C4—C5	1.376 (12)	C20—N3	1.376 (12)
C4—H4	0.9300	C20—H20	0.9300
C5—N4	1.331 (10)	N2—N3	1.304 (9)
C5—C6	1.494 (13)	N3—H3A	0.97 (4)
C6—N5	1.331 (9)	N5—N6	1.376 (9)
C6—C7	1.363 (12)	N6—H6A	0.98 (16)
C7—C8	1.362 (15)	O2—Ni1^iii^	2.163 (6)
C7—H7	0.9300	O4—Ni1^iii^	2.177 (4)
C8—N6	1.301 (11)	O5—Ni2^iv^	2.142 (4)
C8—H8	0.9300	O7—Ni2^iv^	2.217 (6)
C9—O6	1.227 (9)	O9—H2W	0.82 (4)
C9—O5	1.257 (9)	O9—H1W	0.82 (12)
C9—C10	1.555 (10)	O10—H3W	0.82 (5)
C10—O8	1.251 (8)	O10—H4W	0.82 (4)
C10—O7	1.287 (9)	O11—H6W	0.82 (4)
C11—O1	1.228 (7)	O11—H5W	0.82 (5)
			
O1—Ni1—O2^i^	91.6 (2)	O1—C11—O2	124.2 (7)
O1—Ni1—O4^i^	158.28 (17)	O1—C11—C12	119.4 (6)
O2^i^—Ni1—O4^i^	76.2 (2)	O2—C11—C12	116.3 (6)
O1—Ni1—O3	75.4 (2)	O3—C12—O4	128.8 (8)
O2^i^—Ni1—O3	101.4 (2)	O3—C12—C11	114.9 (7)
O4^i^—Ni1—O3	89.3 (2)	O4—C12—C11	116.3 (7)
O1—Ni1—N2	99.0 (2)	N1—C13—C14	123.2 (9)
O2^i^—Ni1—N2	162.9 (2)	N1—C13—H13	118.4
O4^i^—Ni1—N2	97.4 (2)	C14—C13—H13	118.4
O3—Ni1—N2	94.3 (2)	C15—C14—C13	118.2 (9)
O1—Ni1—N1	100.5 (2)	C15—C14—H14	120.9
O2^i^—Ni1—N1	91.3 (2)	C13—C14—H14	120.9
O4^i^—Ni1—N1	97.8 (2)	C16—C15—C14	121.9 (8)
O3—Ni1—N1	166.7 (2)	C16—C15—H15	119.0
N2—Ni1—N1	73.7 (2)	C14—C15—H15	119.1
O5^ii^—Ni2—O6	91.0 (2)	C15—C16—C17	118.8 (9)
O5^ii^—Ni2—O8	157.52 (16)	C15—C16—H16	120.6
O6—Ni2—O8	76.1 (2)	C17—C16—H16	120.6
O5^ii^—Ni2—N5	100.1 (2)	N1—C17—C16	121.0 (8)
O6—Ni2—N5	162.0 (2)	N1—C17—C18	114.2 (7)
O8—Ni2—N5	97.3 (2)	C16—C17—C18	124.6 (8)
O5^ii^—Ni2—O7^ii^	76.95 (19)	N2—C18—C17	121.7 (7)
O6—Ni2—O7^ii^	104.2 (2)	N2—C18—C19	107.2 (8)
O8—Ni2—O7^ii^	88.3 (2)	C17—C18—C19	131.2 (8)
N5—Ni2—O7^ii^	92.1 (2)	C20—C19—C18	106.3 (8)
O5^ii^—Ni2—N4	99.5 (2)	C20—C19—H19	126.8
O6—Ni2—N4	91.5 (2)	C18—C19—H19	126.9
O8—Ni2—N4	99.2 (2)	C19—C20—N3	108.0 (9)
N5—Ni2—N4	72.9 (2)	C19—C20—H20	126.0
O7^ii^—Ni2—N4	163.9 (2)	N3—C20—H20	126.0
N4—C1—C2	122.3 (8)	C13—N1—C17	116.6 (7)
N4—C1—H1	118.9	C13—N1—Ni1	127.5 (6)
C2—C1—H1	118.9	C17—N1—Ni1	115.8 (5)
C3—C2—C1	119.2 (9)	N3—N2—C18	108.4 (7)
C3—C2—H2	120.4	N3—N2—Ni1	136.9 (5)
C1—C2—H2	120.4	C18—N2—Ni1	114.3 (5)
C2—C3—C4	118.7 (8)	N2—N3—C20	109.9 (8)
C2—C3—H3	120.6	N2—N3—H3A	118 (3)
C4—C3—H3	120.6	C20—N3—H3A	132 (3)
C5—C4—C3	118.9 (9)	C5—N4—C1	118.6 (7)
C5—C4—H4	120.6	C5—N4—Ni2	115.7 (5)
C3—C4—H4	120.6	C1—N4—Ni2	125.6 (5)
N4—C5—C4	122.0 (9)	C6—N5—N6	102.3 (6)
N4—C5—C6	116.6 (7)	C6—N5—Ni2	119.1 (5)
C4—C5—C6	121.2 (8)	N6—N5—Ni2	138.5 (5)
N5—C6—C7	113.3 (8)	C8—N6—N5	111.7 (7)
N5—C6—C5	115.6 (7)	C8—N6—H6A	123 (10)
C7—C6—C5	131.1 (8)	N5—N6—H6A	116 (10)
C6—C7—C8	103.7 (8)	C11—O1—Ni1	114.3 (4)
C6—C7—H7	128.1	C11—O2—Ni1^iii^	114.2 (5)
C8—C7—H7	128.1	C12—O3—Ni1	116.1 (6)
N6—C8—C7	108.9 (7)	C12—O4—Ni1^iii^	116.1 (5)
N6—C8—H8	125.6	C9—O5—Ni2^iv^	117.1 (4)
C7—C8—H8	125.6	C9—O6—Ni2	116.4 (6)
O6—C9—O5	128.1 (7)	C10—O7—Ni2^iv^	111.8 (5)
O6—C9—C10	116.2 (7)	C10—O8—Ni2	113.6 (4)
O5—C9—C10	115.6 (6)	H2W—O9—H1W	116 (8)
O8—C10—O7	123.8 (8)	H3W—O10—H4W	115 (5)
O8—C10—C9	117.7 (6)	H6W—O11—H5W	114 (6)
O7—C10—C9	118.5 (6)		

Symmetry codes: (i) *x*−1/2, −*y*+5/2, *z*; (ii) *x*+1/2, −*y*−1/2, *z*; (iii) *x*+1/2, −*y*+5/2, *z*; (iv) *x*−1/2, −*y*−1/2, *z*.

### Hydrogen-bond geometry (Å, °)

**Table:**

*D*—H···*A*	*D*—H	H···*A*	*D*···*A*	*D*—H···*A*
N3—H3A···O11^v^	0.97 (4)	1.81 (2)	2.745 (10)	160 (5)
N6—H6A···O10^vi^	0.98 (16)	2.0 (2)	2.732 (9)	133 (5)
O9—H2W···O7^vii^	0.82 (4)	2.02 (4)	2.822 (9)	166 (6)
O9—H1W···O11^viii^	0.82 (12)	2.22 (15)	2.813 (11)	130 (16)
O10—H3W···O4^ix^	0.82 (5)	2.30 (7)	2.834 (9)	123 (7)
O10—H4W···O9^viii^	0.82 (4)	2.08 (3)	2.811 (11)	148 (5)
O11—H5W···O9^x^	0.82 (5)	2.15 (4)	2.813 (11)	138 (5)
O11—H6W···O8^xi^	0.82 (4)	2.17 (4)	2.876 (9)	144 (6)

Symmetry codes: (v) −*x*+1, −*y*+2, *z*+1/2; (vi) *x*, *y*−1, *z*; (vii) *x*+1/2, −*y*+1/2, *z*; (viii) *x*+1/2, −*y*+3/2, *z*; (ix) −*x*+3/2, *y*−1/2, *z*−1/2; (x) *x*−1/2, −*y*+3/2, *z*; (xi) *x*, *y*+1, *z*.
